# Over-the-counter Antipyretics Use Among Children from Southeastern Poland

**DOI:** 10.34763/jmotherandchild.20212501.d-20-00024

**Published:** 2021-10-11

**Authors:** Olga Pyznar, Nina Mól, Magdalena Zasada, Wojciech Zasada, Małgorzata Mazurek, Przemko Kwinta

**Affiliations:** 1 Department of Paediatrics, University Children’s Hospital, Krakow, Poland; 2 Department of Paediatrics, Faculty of Medicine, Jagiellonian University Medical College, Krakow, Poland; 3 Institute of Cardiology, Faculty of Medicine, Jagiellonian University Medical College, Krakow, Poland

**Keywords:** over-the-counter, antipyretics, parents, children, factors, sources

## Abstract

**Background:**

Over-the-counter (OTC) drugs are becoming increasingly popular. However, little is known about parents’ practices concerning the use of OTC antipyretics in children. This paper aimed to study the habits and knowledge of parents regarding the use of OTC antipyretics in their offspring, considering the demographic and socioeconomic characteristics of the families.

**Material and methods:**

A multiple-purpose survey was conducted anonymously among 229 parents of patients hospitalised in the Department of Paediatrics, University Children’s Hospital, Krakow. Each parent answered 23 questions regarding OTC antipyretics use in his/her hospitalised child throughout the whole child’s life. The data was statistically analysed.

**Results:**

OTC antipyretics are administered to their children by 92% of parents. In the vast majority (87%), health care professionals or a leaflet were the sources of information on the drug and its dosage. Parents also used information from TV or the Internet (27%) and friends and family (30%), especially those in the younger age group. Families with high socioeconomic status were more likely to use health care professionals’ advice for drug knowledge. Parents of children with allergic diseases made less use of nonmedical sources of knowledge.

**Conclusions:**

The majority of parents use OTC antipyretic drugs in their children. However, a high percentage of people using nonmedical sources of information is of concern. It is necessary to educate caregivers and to build the parents’ awareness that they take an active role in their child’s treatment. It would be useful to create generally available recommendations for home treatment.

## Introduction

The market for over-the-counter drugs in Poland is very large. Taking into account the entire pharmaceutical sector, in 2019 OTC’s market share amounted to 32%. Moreover, in recent years, there has been a gradual increase in the sales value of OTC drugs, and in 2019, the increase in their sales in Poland was higher than the global growth of the OTC drugs market. Among this group of products, antipyretics form the fourth-largest group of drugs sold, and their sales are estimated at PLN 1.8 billion per year [1]. In the Polish population, use of OTC drugs is the largest in the paediatric group. It accounts for approximately 29–32% of administered medications [2]. The wide use and availability of OTC drugs is the result of assumptions by the World Health Organisation that they give many benefits to patients: they enable the faster acquisition of the drug, broaden the availability of various forms of products, and make the parent take an active part in the treatment of the child. Moreover, by reducing the number of physician’s office visits, OTC drug use benefits the health care system and facilitates access to physicians for patients with chronic conditions [3]. However, the administration of over-the-counter antipyretics requires basic knowledge about the dosage and indications for these drugs.

Over-the-counter antipyretics available in Poland include paracetamol, ibuprofen, metamizole and acetylsalicylic acid. The first two form the vast majority of such medicines administered to children [4]. Their inappropriate use can be associated with the lack of therapeutic effect when the dose of the drug is too small, and overdosing can result in life-threatening conditions such as liver or kidney failure [5,6]. Until now, the data on the use of over-the-counter antipyretics in the paediatric population in Poland was limited. Żołnierczuk-Kieliszek et al. [7] showed that antipyretic drugs are the third most common, after antitussives and sore throat medications, among OTC drugs administered by parents to their offspring. In turn, Łoś-Rycharska et al. showed the physician’s overarching role in advising parents on the choice of the type of antipyretic drug for their child, as well as on the correct dosage [8]. Interestingly, available publications show that people under and over 30 years of age make different choices when it comes to using over-the-counter dietary supplements [9,10], in particular antipyretic drugs [8], both in terms of their choice for themselves and for their offspring.

The objectives of this study were to estimate the habits of OTC antipyretics use in a paediatric population by the parents and to examine familial, social and parents’ demographic factors associated with their use, with particular emphasis on the parents’ age and sources of knowledge about OTC antipyretics use.

## Methods

A multiple-purpose survey was conducted among parents of patients hospitalised in the Children’s Disease Clinic of the University Children’s Hospital in Krakow in the period from January to October 2018. This hospital hospitalises children up to 18 years of age from southeastern Poland, mainly from the Lesser Poland Voivodeship. Patients were hospitalised due to childhood infectious diseases, such as upper and lower respiratory tract infections, gastrointestinal tract infections and urinary tract infections. Parents staying with their child in the ward were asked to complete a questionnaire on the use of OTC antipyretics for their currently hospitalised child. They were orally informed by the investigator about completing the questionnaire based on their experience with the use of OTC antipyretics in relation to the child who is currently hospitalised. They were asked to consider the entire period of the child’s life. The first part of the survey concerned the general characteristics of the hospitalised child and his/her parent (e.g., age, level of education, family socioeconomic status [SES]), and the second part included questions about the hospitalised child’s use of OTC antipyretics and the parent’s sources of knowledge about them (Appendix 1). Socioeconomic status (SES) was based on the parent’s subjective assessment. Before the survey was conducted, the consent of the relevant Bioethics Committee (No. 1072.6120.6.2018) and the consent of the patient’s parent were obtained. The categorical variables were compared by Pearson’s chi-squared test or Fisher’s exact test if 20% of cells had an expected count of less than 5. Two-sided P-values < 0.05 were considered statistically significant. All calculations were performed with JMP®, Version 15.2 (SAS Institute Inc., Cary, NC, USA).

## Results

Of the 229 parents surveyed, 197 (86 %) were mothers. The mean parent age was 32.1 years (SD 6.2 years [range 18–54 years]). The number of children per parent varied from 1 to 6, with a median of 2 . The detailed data regarding parent’s demographic information is summarised in Table 1 and Table 2.

**Table 1:** Demographic characteristics of parents (N=229)

	n (%)
Number of children (below 18 yr)	
**1**	102 (44.5%)
**2**	91 (39.8%)
**3+**	36 (15.7%)
**Place of living**	
**Rural area**	81 (35.4%)
**City < 10,000 population**	12 (5.2%)
**City 10,000-100,000 population**	28 (12.2%)
**City > 100,000 population**	107 (46.7%)
**Subjective family socioeconomic status**	
**High**	158 (69.0%)
**Moderate**	69 (30.1%)
**Low**	2 (0,9%)
**Education: mother**	
**Primary**	7 (3.1%)
**Secondary**	75 (32.7%)
**Bachelor**	11 (4.8%)
**High**	136 (59.4%)
**Education: father**	
**Primary**	13 (5.7%)
**Secondary**	105 (45.9%)
**Bachelor**	8 (3.5)
**High**	102 (44.5%)
**Parent’s age**	
**≤ 30**	94 (42%)
**>30**	131 (58%)

**Table 2:** Socio-demographic and personal characteristics of children hospitalised in the Department of Paediatrics, University Children’s Hospital in Krakow, taking OTC antipyretic drugs

	Children using OTC antipyretics n (%)	All children in the study n
Total no. of participants	178	227
Child’s gender		
**Males**	97 (54.8%)	121 (53.3%)
**Females**	79 (44.6%)	105 (46.3%)
Prematurity	21 (12%)	31 (13.8%)
Delivery		
**1**	83 (50.6%)	115 (54.2%)
**2**	61 (37.2%)	71 (33.5%)
**3+**	20 (12.2%)	26 (12.3%)
**Average grams (SD) birth weight;**	3234(+/- 609)	3227(+/-697)
Chronic diseases	26 (15.1%)	34 (15.3%)
**Allergic diseases**	38 (21.3%)	45 (19,8%)

### Frequency of OTC antipyretics use

Almost 80% of parents admitted to administering OTC antipyretic drugs to their children. When the question concerned a particular medicinal product, this tendency became higher: 85% of parents (193 from 226) administer paracetamol, and 80% of parents administer ibuprofen (181 from 227) to their children. Taking both of these responses into account, 73% of parents use both paracetamol and ibuprofen, 12% use paracetamol only, 7% only ibuprofen and 8% of parents do not use any of these OTC antipyretic drugs (Table 3).

**Table 3:** Frequency and motives of administering OTC antipyretic drugs to children by their parents: analysis regarding parent’s age

	Age ≤ 30 years	Age > 30 years	P and test used
	(n=90)	(n=126)	
Use of OTC antipyretics	63 (70%)	106 (84%)	0.0315^p^
Frequency of using OTC: paracetamol			
**When required (pain, fever)**	72 (80%)	108 (86%)	0.2990^p^
**Once a week**	0	0	
**Once a day**	1 (1%)	0	
**I do not use**	16 (18%)	17 (14%)	
**Frequency of using OTC: ibuprofen**			
**When required (pain, fever)**	62 (69%)	108 (86%)	0.0159^p^*
**Once a week**	0	0	
**Once a day**	1 (1%)	0	
**I do not use**	26 (29%)	18 (14%)	
**Reason for administering OTC antipyretics**			
**Pain**	17 (19%)	44 (35%)	0.0089^p^*
**Fever**	70 (78%)	111 (89%)	0.0289^p^*
**Doctor’s advice**	40 (44%)	66 (53%)	0.2267^p^
**Common cold/flu**	21 (23%)	34 (27%)	0.5544^p^

p Pearson chi square test * Difference statistically significant

### Reasons for OTC antipyretics use

Almost 84% of parents pointed out fever as a reason to use antipyretics, which was also the most common response when parents were asked about the most frequent indication for administering the OTC antipyretic drugs to children. They also admitted to using these drugs when their children had fever: 49% due to a physician’s advice, 28% to alleviate pain and 25% to treat a common cold/flu.

### Places to buy OTC antipyretics

The most common place where the parents buy OTC antipyretic drugs is a pharmacy (95% of parents). Places such as general stores or petrol stations are very rarely specified as a source for buying this drug.

### Sources of knowledge about OTC antipyretic drugs

According to the parents, the most common source of health risk information regarding OTC antipyretic drugs is the health care provider (85% of parents). Less common sources are family and friends (for 30% of parents) and the TV and Internet (for 27% of parents).

The two most common sources of information regarding the OTC antipyretic drug dosage are container labels and health care providers (83% and 75%, respectively). The TV/Internet and family/friends very rarely serve as a source of information regarding dosages of OTC antipyretic drugs (8% and 6%, respectively). Interestingly, parents who declared that they search for information on the appropriate OTC antipyretic drug dosage for their children on the Internet/TV or ask their family members/friends about it were significantly younger than those who refused to use these sources of information to find the appropriate OTC antipyretic drug dosage for their children (Tables 4 and 5).

**Table 4:** Sources of information and place of purchase OTC antipyretics - analysis regarding parent’s age.

	Age ≤ 30 years	Age > 30 years	P and test
	(n=90)	(n=126)	used
Place of purchasing OTC antipyretics			
**Pharmacy**	82 (91%)	122 (97%)	0.0707^p^
**General store/ convenience store**	2 (2%)	5 (4%)	0.4750^p^
**Petrol station**	2 (2%)	0	0.0927^p^
**Source of health risk information about OTC antipyretics**			
**Family, friends**	28 (31%)	39 (31%)	0.9802^p^
**TV, Internet**	20 (22%)	38 (30%)	0.1944^p^
**Health care providers**	79 (88%)	104 (83%)	0.2915^p^
**Source of information about OTC antipyretics’ dosage**			
**Container label/ medication insert**	68 (76%)	110 (87%)	0.0254^p^*
**TV, Internet**	13 (14%)	5 (4%)	0.0060^p^*
**Family, friends**	10 (11%)	4 (3%)	0.0195^p^*
**Health care providers**	69 (77%)	94 (75%)	0.7282^p^
**Own knowledge experience and**	6 (7%)	9 (7%)	0.8920^p^

p- Pearson chi square test * - difference statistically significant

**Table 5:** Age of parents, taking into account the preferred source of knowledge regarding the administration of appropriate doses of OTC antipyretic drugs to their children

Where do you get information about the dosage of antipyretic medications for children?			
	Yes	No	p
Container years) label (mean age (SD);	32.52 (6.02)	31.37 (6.86)	0.1471^W^
Internet, TV (mean age (SD); years)	29.60 (5.13)	32.57 (6.22)	0.0319^W^*
Family, years) friends (mean age (SD);	29.47 (7.35)	32.51 (6.06)	0.0425^W^*
Health (SD); years) care providers (mean age	32.11 (6.03)	32.88 (6.62)	0.4817^W^
According to my own experience and knowledge (mean age (SD); years)	34.18 (8.47)	32.15 (5.95)	0.5200^W^

W Mann-Whitney-Wilcoxon test * Difference statistically significant

### Impact of parental SES on OTC antipyretic drug usage

When we divide our population of parents into two subgroups taking into account their socioeconomic status (high vs. moderate/low), we find only one statistically significant difference regarding OTC antipyretic drug usage. The parents from the high socioeconomic status group based their decision to give their child antipyretics on information from a health care provider regarding the health risks more often than did parents from the moderate/low socioeconomic status group (89% vs. 76%, p=0.0219, Fisher’s Exact Test).

### Habits of OTC antipyretics administration to children with allergic diseases

We found a similar situation when we divided our parents into two subgroups according to the presence of allergic disease in their children (present vs. absent). The only significant difference related to choosing family members/friends as a source of information on the scope of health risk from OTC antipyretic drug usage. The parents whose children have allergic diseases use this source significantly more rarely than those with children without allergic disease (11% vs. 34%, p=0.0145, Fisher’s Exact Test). We did not find any significant differences between parents who live in cities and those living in villages.

### Parents’ age and OTC antipyretic administration

Interestingly, 84% of parents over the age of 30 administered OTC antipyretic drugs to their children, in comparison to 70% of parents below the age of 30. Moreover, parents over the age of 30 use the OTC antipyretic drugs more often to treat pain and fever and choose ibuprofen more frequently in comparison to the parents below the age of 30. Additionally, the older parents more frequently use the container label/ drug leaflet as a source of information regarding dosage, whereas the parents below the age of 30 more often search for information on dosage on TV or the Internet or ask their family members or friends (Table 5).

## Discussion

The results of the survey underline that the use of OTC antipyretic drugs in the paediatric population of the Lesser Poland Voivodeship is very common. The percentage of parents administering the aforementioned drugs to their children in that area is comparable to data obtained from other worldwide studies [11,12].

According to the results of our study, about 80% of parents admit that they provide OTC painkillers (paracetamol, ibuprofen) to their children. Interestingly, after giving the trade name of the drug, the number of positive responses increases. It is therefore possible that parents do not always know the group of drugs to which the particular product they administer to their child belongs. Our research has shown that fever remains the most common cause for administering over-the-counter antipyretic drugs (83.8%). The thesis written by Crocetti et al. shows that, despite the passage of years, there is still anxiety about fever among parents, and some parents still believe that untreated fever can lead to brain damage or death [13].

Unfortunately, despite numerous concerns regarding the adverse effects of fever, the parents also use nonmedical sources of information concerning the treatment and administration of OTC drugs to their children. As many as 27% of parents also use the Internet and television as a source of knowledge, and 30% learn from friends and family members. Information obtained in this way is not always reliable and, as the research shows, even parents consider it less useful than other sources [14]. This is confirmed by the results of the research performed in the Serbian population, which showed that parents reading the leaflet are more aware of how to use a drug than those who only read the information on the outer packaging or use the Internet as their source of knowledge [15]. The aforementioned analysis shows that nonmedical sources of knowledge are used significantly less often by parents of children with allergic diseases (11% vs. 34%). This can be related to parents’ awareness of the scope of the possible risk of an allergic reaction if their child takes the drug, the need for increased vigilance, and the willingness to obtain more extensive knowledge about the drugs they choose to administer to the person under their care.

In our thesis, we have shown that health care professionals are the most frequent source of knowledge about drugs used. Similar results are shown by population studies done in other countries [14,15].

The analysis shows that health care professionals’ advice on the use of OTC drugs is significantly more often accepted by people of high SES (89% vs. 76%). The literature includes conclusions contradictory to this result. In Ecklund’s study, we observe that parents belonging to the high SES group are more likely to administer OTC drugs on their own than are parents belonging to the lower SES group [16]. Moreover, some studies show that parents belonging to the high and medium SES group are more likely to more often treat their children using alternating administration of different antipyretic drugs [17].

In the vast majority of cases (83%), the parents use the leaflet to gain knowledge on the drug dosage. However, it is important to recall the studies of groups of American parents, only 40% of whom, despite access to the leaflet and appropriate dosage devices, were able to calculate the appropriate dosage for their child and only 67 % of whom were able to correctly measure the intended quantity [18]. In the thesis written by Pereira et al., it is underlined that as much as 70% of the analgesics administered to children did not reach their therapeutic value [17]. This leads to lower drug efficacy, can increase parental anxiety, and reduces child comfort. It can also increase the number of physician’s office visits, which is contradictory to the original idea of self-treatment and use of OTC drugs.

Another disconcerting conclusion resulting from the aforementioned research is the observed discrepancy in parents’ awareness regarding the group in which the drugs belong. The literature shows that less than half of the parents verify the drug’s active substance before administration [7,11]. The caregivers frequently admit to administering incorrect doses of over-the-counter drugs to their children or to not observing the minimum intervals between subsequent doses [7,14]. Such behaviour can lead to side effects or drug overdose. This should be particularly emphasised, taking into account that accidental or deliberate poisoning with easily available paracetamol remains the most common known cause of acute liver failure in the paediatric population [5].

Data presented in Tables 4 and 5 reveals that a significantly lower average age is observed in the group of parents who acquire knowledge on the drug dosage from the Internet/TV or family members and friends than in the group where no information is obtained from the aforementioned sources. This indicates a certain tendency among younger generations of parents to attach much more importance to knowledge coming from these sources. Due to the progressing digitalisation of life and the significant impact of “social media” on the process of parents’ decision making [19], this impact will probably increase in subsequent years. Therefore, it is very important to promote as knowledge sources those with verified content and at the same time to make that information understandable and accessible to parents using the Internet/TV as a source.

## Limitations of the study

One of the limitations of our study was the lack of multivariate analyses that may possibly show whether the age effect is not apparent and not the result of other characteristics of the studied families. The second drawback is the construction of the questionnaire, in which the overall frequency of drug use was reduced to a simple yes/no division without a time perspective.

## Strengths of the study

The strengths of this study are its large sample size and high response rate.

In addition, our article emphasises the need to educate parents in the use of OTC antipyretics, for example, in terms of simple information about what the drug given to their child is and what it should do. According to the results of our survey, many parents use nonmedical sources of knowledge, and it should be ensured that the content published or presented in these sources is of high quality and accessible to parents.

## Key points

1. OTC antipyretic drugs are commonly administered to children.
2. The paediatricians working in primary health care centres should broadly educate caregivers in the scope of that use and its risks. It is necessary to encourage parents, especially those below the age of 30, to use reliable sources of information about drugs that provide complete knowledge about the drug and potential side effects.
3. General practitioners should also warn parents against overmedicating their children and present situations in which the administration of the drug is necessary.
4. Establishing reliable and publicly available recommendations for parents on the use of these drugs and making that information also available on the Internet, especially in social media, would facilitate treatment in which the parents play an increasingly important role.
5. It is necessary to perform further broader analyses regarding the Polish population to objectively determine whether parents can take care of their febrile children properly and to verify their knowledge of drugs they willingly administer to their children.

## Abbreviations

OTC: over-the-counter
SES: socioeconomic status

## Appendix 1

***Assessment of over-the-counter antipyretics (OTC) among child population: prospective cohort study***

1. Gender of the person completing the survey:

a) Female

b) Male

2. Age of the person completing the survey: ...... years

3. Number of children: .....

4. Place of living:

a) Rural area

b) City <10 thousand

c) City of 10–100 thousand

d) City >100 thousand

5. Mother’s education:

a) Primary

b) Secondary

c) Bachelor

d) High

6. Father’s education:

a) Primary

b) Secondary

c) Bachelor

d) High

7. How do you assess your material situation:

a) High

b) Moderate

c) Low

8. Number of rooms in the house/apartment (rooms +kitchen): ....

9. Date of birth of the child: ................

10. Gender of the child:

a) Female

b) Male

11. A hospitalized baby is from: …… pregnancy, …… delivery

12. Birth weight: ..............

13. Is your baby born prematurely?

a) Yes (....... week of pregnancy)

b) No

14. Does your child have any chronic diseases?

a) Yes, which ones? ......................................................

b) No

15. Does your child have any of the following diseases?

a) Atopic dermatitis

b) Bronchial asthma

c) Allergic rhinitis

d) Allergic conjunctivitis

e) Other allergic disease; which one? ...........................

16. Have you ever given any over-the-counter antipyretics to your child?

a) Yes

b) No

17. How often do you administer paracetamol (e.g., Apap, Efferalgan, Pedicetamol) to your child?

a) If necessary (e.g., pain, fever)

b) Once a week

c) Once a day

d) I do not use

e) Other; which ones? .................................................

18. How often do you administer ibuprofen (e.g., Ibum, Nurofen, Ibufen) to your child?

a) If necessary (e.g., pain, fever)

b) Once a week

c) Once a day

d) I do not use

e) Other, which ones? .................................................

19. Which of the following preparations do you administer to your child? (You can select more than one response)

a) Apap junior

b) Efferalgan suppository

c) Ibufen junior

d) Ibufen for children raspberry/strawberry forte

e) Ibum junior banana/raspberry

f) Nurofen for children orange/strawberry forte

g) Nurofen for junior children

h) Paracetamol hermina

i) Paracetamol hasco

j) Pedicetamol

k) Other; which ones? ..................................................

20. Why do you administer your child antypyretics? (You can select more than one response)

a) The child reports pain

b) Fever

c) Doctor’s recommendations

d) Cold/illness

e) Other; which ones? ..................................................

21. Where do you purchase antipyretics? (You can select more than one response)

a) Pharmacy

b) Grocery store

c) Service station

d) Other; which ones? .................................................

22. How do you get information about possible side effects of antipyretics? (You can select more than one response)

a) Family, friends

b) TV, Internet

c) Health care professionals

d) Other .........................................................................

23. How do you get information about the dosage of antipyretics for your child? (You can select more than one response)

a) Container label/ medication insert

b) Internet, TV

c) Family, friends

d) Health care professionals

e) According to my own experience and knowledge
